# Increased Expression of the Neuropeptides PACAP/VIP in the Brain of Mice with CNS Targeted Production of IL-6 Is Mediated in Part by Trans-Signalling

**DOI:** 10.3390/ijms25179453

**Published:** 2024-08-30

**Authors:** Alessandro Castorina, Jurgen Scheller, Kevin A. Keay, Rubina Marzagalli, Stefan Rose-John, Iain L. Campbell

**Affiliations:** 1 Laboratory of Cellular and Molecular Neuroscience, School of Life Sciences, Faculty of Science, University of Technology Sydney, Sydney, NSW 2007, Australia; rubina.marzagalli@uts.edu.au; 2 Institute of Biochemistry and Molecular Biology II, Medical Faculty, Heinrich-Heine University, 40225 Düsseldorf, Germany; jscheller@uni-duesseldorf.de; 3 Discipline of Anatomy and Histology, School of Medical Sciences, The University of Sydney, Sydney, NSW 2006, Australia; kevin.keay@sydney.edu.au; 4 Institute of Biochemistry, Medical Faculty, Christian Albrechts University, 24098 Kiel, Germany; rosejohn@biochem.uni-kiel.de; 5 School of Molecular Bioscience, University of Sydney, Sydney, NSW 2006, Australia; iain.campbell@sydney.edu.au

**Keywords:** interleukin 6, cytokine, PACAP, VIP, gp130, trans-signalling, neuroinflammation, astrocytes

## Abstract

Inflammation with expression of interleukin 6 (IL-6) in the central nervous system (CNS) occurs in several neurodegenerative/neuroinflammatory conditions and may cause neurochemical changes to endogenous neuroprotective systems. Pituitary adenylate cyclase-activating polypeptide (PACAP) and vasoactive intestinal polypeptide (VIP) are two neuropeptides with well-established protective and anti-inflammatory properties. Yet, whether PACAP and VIP levels are altered in mice with CNS-restricted, astrocyte-targeted production of IL-6 (GFAP-IL6) remains unknown. In this study, PACAP/VIP levels were assessed in the brain of GFAP-IL6 mice. In addition, we utilised bi-genic GFAP-IL6 mice carrying the human sgp130-Fc transgene (termed GFAP-IL6/sgp130Fc mice) to determine whether trans-signalling inhibition rescued PACAP/VIP changes in the CNS. Transcripts and protein levels of PACAP and VIP, as well as their receptors PAC1, VPAC1 and VPAC2, were significantly increased in the cerebrum and cerebellum of GFAP-IL6 mice vs. wild type (WT) littermates. These results were paralleled by a robust activation of the JAK/STAT3, NF-κB and ERK1/2MAPK pathways in GFAP-IL6 mice. In contrast, co-expression of sgp130Fc in GFAP-IL6/sgp130Fc mice reduced VIP expression and activation of STAT3 and NF-κB pathways, but it failed to rescue PACAP, PACAP/VIP receptors and Erk1/2MAPK phosphorylation. We conclude that forced expression of IL-6 in astrocytes induces the activation of the PACAP/VIP neuropeptide system in the brain, which is only partly modulated upon IL-6 trans-signalling inhibition. Increased expression of PACAP/VIP neuropeptides and receptors may represent a homeostatic response of the CNS to an uncontrolled IL-6 synthesis and its neuroinflammatory consequences.

## 1. Introduction

Interleukin 6 (IL-6) is a prototypical cytokine founder member of the IL-6 family, a group of structurally related cytokines, which include IL-6, IL-11, IL-27, IL-31, leukaemia inhibitory factor, oncostatin M, cardiotrophin-1, neuropoietin, cardiotrophin-like cytokine and two viral analogues of IL-6 [1,2,3]. The IL-6 family of cytokines bind to class I cytokine receptors, including IL-6Rα, which are membrane proteins without intrinsic enzymatic activity. Due to the lack of activity, IL-6 recruits additional receptor proteins shared with other cytokines to signal, namely gp130. In this respect, the IL-6 family is often referred to as the gp130 family of cytokines. IL-6 signalling occurs either through the binding of the cytokine to the canonical membrane-anchored IL-6R (classic signalling) or, alternatively, through the formation of a biologically active complex with a shed soluble extracellular domain of IL-6R (sIL-6R). The IL-6/sIL-6R complex, which can oligomerise with gp130 to transduce IL-6 signals to cells that do not normally express surface-bound IL-6Rs, is referred to as trans-signalling [4]. Interestingly, evidence from these laboratories have shown that trans-signalling is a dominant mechanism for the pathogenic actions of IL-6 in the brain [5] and is mostly blocked in the presence of the natural occurring soluble form of the gp130 protein subunit (sgp130Fc) [6].

IL-6 is a major pro-inflammatory cytokine that plays important biological functions as an effector molecule of the immune system, both in the central nervous system (CNS) and in the periphery [7,8]. Local IL-6 production is beneficial in the acute phases of CNS injury and promotes healing by decreasing neuronal apoptosis and oxidative damage [9]. Conversely, persistent IL-6 produces deteriorating effects, especially in subcortical regions and in the cerebellum, as demonstrated in studies using transgenic mice with astrocyte-targeted production of IL-6 (GFAP-IL6) [10]. GFAP-IL6 mice show overt signs of cognitive decline [11], abnormal proliferative angiopathy and blood–brain barrier breakdown [12], as well as astro- and microgliosis and other neuropathological alterations compatible with a state of chronic degenerative/inflammatory encephalopathy [11]. So far, numerous molecular signatures of neuroinflammation have been described in the CNS of these transgenic mice [5]; however, to our knowledge, no one has ever investigated if and how endogenous neuroprotective peptides are affected by astrocyte-restricted chronic IL-6 production.

Pituitary adenylate cyclase-activating polypeptide (PACAP) and vasoactive intestinal polypeptide (VIP)—two highly homologous peptides—are neuroprotective in several models of CNS disease [13,14,15] and exhibit potent anti-inflammatory properties [16,17,18]. PACAP and VIP activities are mediated by a subgroup of G protein-coupled receptors named VPAC type, which include two receptors (VPAC1 and VPAC2), both of which show similar high affinity for both peptides. Another receptor subtype, termed PAC1, is highly specific for PACAP and comprises at least eight different splicing isoforms. Both VPAC and PAC1 receptor subtypes primarily activate the cAMP/PKA signalling pathways, but they can also trigger other intracellular cascades, depending on the cell type or the nature of the insult, to trigger different cellular responses [19]. Using in vitro models of inflammation, attempts have been made to identify a potential link between these two immunomodulatory agents and IL-6. It was discovered that PACAP and VIP elicits cell-specific regulatory functions on IL-6 secretion by resident glia, acting either as potent stimulators in astrocytes [20] or as inhibitors in polarised microglia [21].

In the present study, bearing in mind the neuropathological consequences of GFAP-IL6 mice and the divergent responses of resident glia to PACAP or VIP, we sought to investigate whether the endogenous levels of the two neuropeptides are affected in these transgenic mice, with the aim of identifying a functional link between IL-6 and neuropeptides levels in the CNS. In addition, given the relevant role of trans-signalling in the pathogenic actions of IL-6, we also explored if co-expression of the natural IL-6 inhibitor (sgp130Fc) in GFAP-IL6 transgenic mice (bi-genic GFAP-IL6/sgp130Fc mice) would prevent IL-6 driven alterations of the peptides’ expression and associated inflammatory pathways.

## 2. Results

### 2.1. PACAP and VIP Levels Are Increased in the Brain of GFAP-IL6 Mice and Partly Reduced by Inhibition of Trans-Signalling

The main purpose of these studies was as follows: (1) to investigate whether the endogenous levels of PACAP and VIP in the cerebrum and cerebellum were altered by the forced production of IL-6 by astrocytes, and (2) to examine if the blockade of trans-signalling prevented such changes. To this end, PACAP and VIP levels were analysed by ELISA in lysates of the cerebrum or cerebellum obtained from WT and GFAP-IL6/sgp130Fc mice. It has previously been reported that transgene-encoded IL-6 levels are higher in the cerebellum than the cerebrum of the GFAP-IL6 mice [22], which allowed us to investigate the dose-dependence of any IL-6 responses in the brain of transgenic mice.

**Figure 1:**
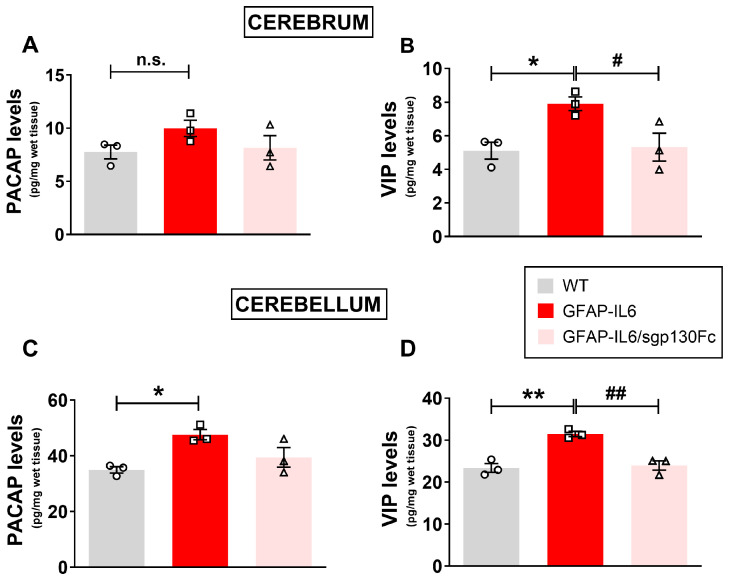
Enhanced PACAP and VIP levels in the cerebrum and cerebellum of GFAP-IL6 mice are partly rescued in bi-genic GFAP-IL6/sgp130Fc mice. PACAP and VIP protein concentrations in lysates from cerebra and cerebella of age-matched six-months-old wild-type (WT), monogenic GFAP-IL6 and bi-genic GFAP-IL6/sgp130Fc mice were determined using commercially available PACAP and VIP mouse ELISA Kits (for details refer to Section 4). Comparisons among WTs, monogenic and bi-genic mice groups (n = 3 per group) were conducted to assess the levels of PACAP or VIP in the cerebrum (**A**,**B**) and cerebellum (**C**,**D**). * *p* < 0.05 or ** *p* < 0.01 vs. WT. # *p* < 0.05 or ## *p* < 0.01 vs. GFAP-IL6 mice. n.s. = not significant. Tukey post-hoc test after analysis of variance.

PACAP levels were slightly increased in the cerebrum of GFAP-IL6 mice compared with WTs, although the increase was not statistically significant (*p* > 0.05 vs. WTs, Figure 1A). Despite the modest increase, co-production of sgp130Fc was sufficient to prevent the PACAP increase in the cerebrum (Figure 1A). In contrast, VIP was significantly increased in GFAP-IL6 mice as compared with WT mice (Figure 1B, *F*_2,8_ = 6.568, * *p* < 0.05 vs. WT). A blockade of trans-signalling in GFAP-IL6/sgp130Fc mice also significantly reduced VIP-induction caused by chronic IL-6 production (**#** *p* < 0.05 vs. GFAP-IL6).

In the cerebellum of WT mice, basal levels of both PACAP and VIP were markedly higher (about 5-fold) than in the cerebrum (Figure 1C,D). Additionally, GFAP-IL6 mice exhibited significantly higher levels of both PACAP (Figure 1C, *F*_2,8_ = 7.147, * *p* < 0.05 vs. WT) and VIP than in WT controls (Figure 1D, *F*_2,8_ = 22.48, ** *p* < 0.01 vs. WT). Like the cerebrum, co-production of sgp130Fc slightly attenuated the increased PACAP (not significant vs. GFAP-IL6) and strongly reduced VIP in the cerebellum of bi-genic GFAP-IL6/sgp130Fc mice (Figure 1D, ## *p* < 0.01 vs. GFAP-IL6).

### 2.2. Trans-Signalling Inhibition Prevents STAT3, NF-κB but Not Erk1/2MAPK Phosphorylation in GFAP-IL6 Mice

**Figure 2:**
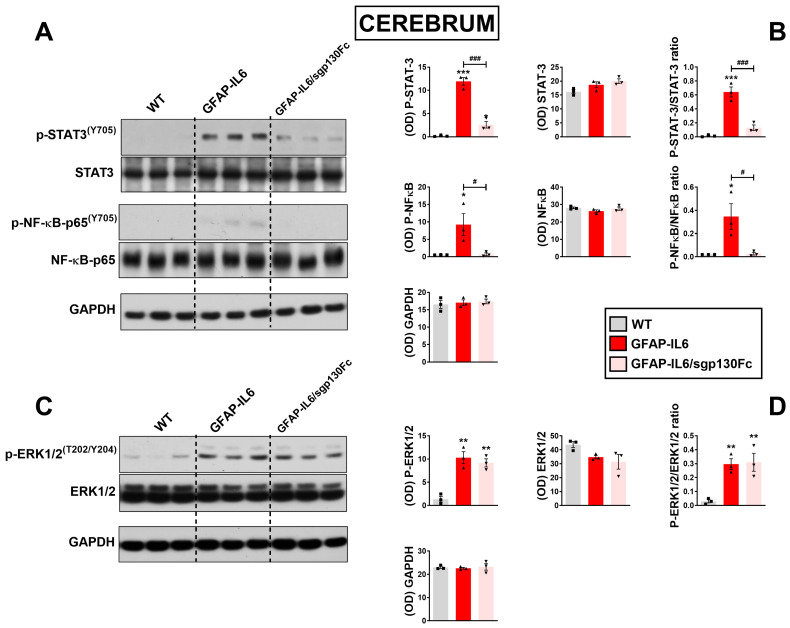
Comparative analyses of STAT3, NFκB and ERK1/2^MAPK^ phosphorylation in the cerebrum of GFAP-IL6 and GFAP-IL6/sgp130Fc mice. Co-expression of sgp130Fc reduces steady-state p-STAT3^(Y705)^ and p-NFκB^(S536)^ but not p-ERK1/2^MAPK^ in the cerebrum of GFAP-IL6 mice. (**A**,**C**) Tissue lysates (15 μg protein per lane) from cerebra of 6-month-old mice were subjected to SDS-PAGE followed by immunoblotting. (**B**,**D**), X-ray films were quantified by densitometry (OD) using NIH ImageJ software (version 1.52) in (**B**) for p-STAT3, STAT3, p-NFκB, NFκB or reported as a ratio between phospho-specific and pan proteins and in (**D**) for p-ERK1/2, ERK1/2 or as a ratio. GAPDH was used as loading control. Values represent the mean ± SEM with n = 3 brains per genotype. * *p* < 0.05, ** *p* < 0.01 or *** *p* < 0.001 vs. WT. # *p* < 0.05 or ### *p* < 0.001 vs. GFAP-IL6 mice. One-Way ANOVA followed Tukey post-hoc test.

These studies were performed to determine which signalling pathways were activated in response to chronic IL-6 and assess if trans-signalling inhibition prevented these effects. Densitometric analyses of Western blot bands revealed a significant increase in phospho-STAT3/STAT3 ratio in GFAP-IL6 mice cerebra with respect to WTs (Figure 2A,B, *F*_2,8_ = 95.45, *** *p* < 0.001 vs. WT). STAT3 activation was strongly reduced in GFAP-IL6/sgp130Fc mice (### *p* < 0.001 vs. WT), and although residual but still significant, phospho-STAT3 persisted in double transgenic mice (* *p* < 0.05 vs. WT).

The nuclear transcription factor NF-κB represents a major transcriptional regulator for numerous pro-inflammatory cytokines, including IL-6 [23]. In physiological conditions, NF-κB is sequestered in the cytoplasm by the IκBα protein. Upon phosphorylation by the kinase IKK, IκBα is rapidly degraded by the proteasome, allowing NF-κB to translocate to the nucleus, where it exerts transcriptional activity [24]. Here, we found that a minor but significant phosphorylation of the p65 subunit of NF-κB was present in GFAP-IL6 mice cerebra (*F*_2,8_ = 8.464, * *p* < 0.05 vs. WT), which was abrogated in GFAP-IL6/sgp130Fc mice (*# p* < 0.05 vs. GFAP-IL6). Phospho-levels of the extracellular regulated protein kinase 1/2 (ERK 1/2) were also influenced by IL-6 overproduction, with increased phospho-ERK1/2 levels in the cerebrum of GFAP-IL6 mice (Figure 2C,D, *F*_2,8_ = 13.03, ** *p* < 0.01 vs. WT); however, levels were not reduced in GFAP-IL6/sgp130Fc mice (** *p* < 0.01 vs. WT; not significant vs. GFAP-IL6), corroborating the idea that activation of the mitogen activated protein kinase (MAPK) pathway likely occurs via the IL-6 canonical signalling pathway.

**Figure 3:**
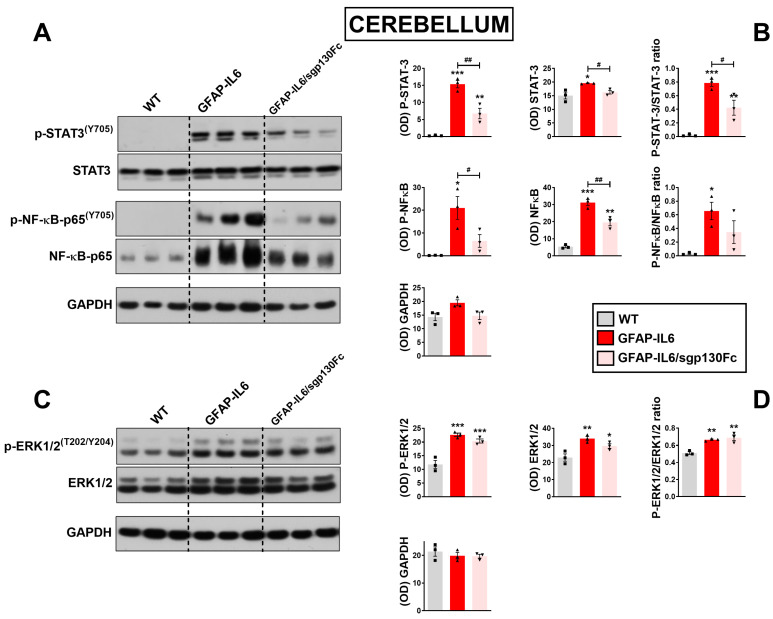
Comparative analyses of STAT3, NFκB and ERK1/2^MAPK^ phosphorylation in the cerebellum of GFAP-IL6 and GFAP-IL6/sgp130Fc mice. Co-expression of sgp130Fc reduces steady-state p-STAT3^(Y705)^ and p-NFκB^(S536)^ but not p-ERK1/2^MAPK^ in the cerebellum of GFAP-IL6 mice. (**A**,**C**) Tissue lysates (15 μg protein per lane) from cerebra of 6-month-old mice were subjected to SDS-PAGE followed by immunoblotting (**B**,**D**). X-ray films were quantified by densitometry (OD) using NIH ImageJ software in (**B**) for p-STAT3, STAT3, p-NFκB and NFκB or reported as a ratio between phospho-specific and pan proteins and in (**D**) for p-ERK1/2 and ERK1/2 or as a ratio. GAPDH was used as a loading control. Values represent the mean ± SEM, with n = 3 brains per genotype. * *p* < 0.05, ** *p* < 0.01 or *** *p* < 0.001 vs. WT. # *p* < 0.05 or ## *p* < 0.01 vs. GFAP-IL6 mice. One-Way ANOVA followed Tukey post-hoc test.

In the cerebellum, phospho-STAT3/STAT3 ratio was increased in GFAP-IL6 mice (Figure 3A,B, *F*_2,8_ = 48.13, *** *p* < 0.001 vs. WT) and partially diminished upon trans-signalling blockage (# *p* < 0.05 vs. *GFAP-IL6*), although retaining moderate transcription factor activity when compared with WTs (** *p* < 0.01 vs. WT). NF-κB phospho-state levels in the cerebellum, measured as a ratio of phospho-NF-κB over total NF-κB, were also increased in monogenic mice carrying the IL-6 transgene (Figure 3A,B, *F*_2,8_ = 6.671, * *p* < 0.05 vs. WT), but could only in part be prevented by co-expression of the sgp130Fc transgene (not significant vs. GFAP-IL6). Consistent with data obtained in the cerebrum, our results showed that phospho-ERK1/2 expression were similarly increased in GFAP-IL6 (Figure 3C,D, *F*_2,8_ = 21.38, ** *p* < 0.01 vs. WT) and GFAP-IL6/sgp130Fc mice compared with WTs (** *p* < 0.01 vs. WT) and were not different between each other (not significant vs. GFAP-IL6).

### 2.3. Forced Astrocyte-Restricted IL-6 Production Increases the PAC1 and VPAC2 Receptors in the Mouse Brain

**Figure 4:**
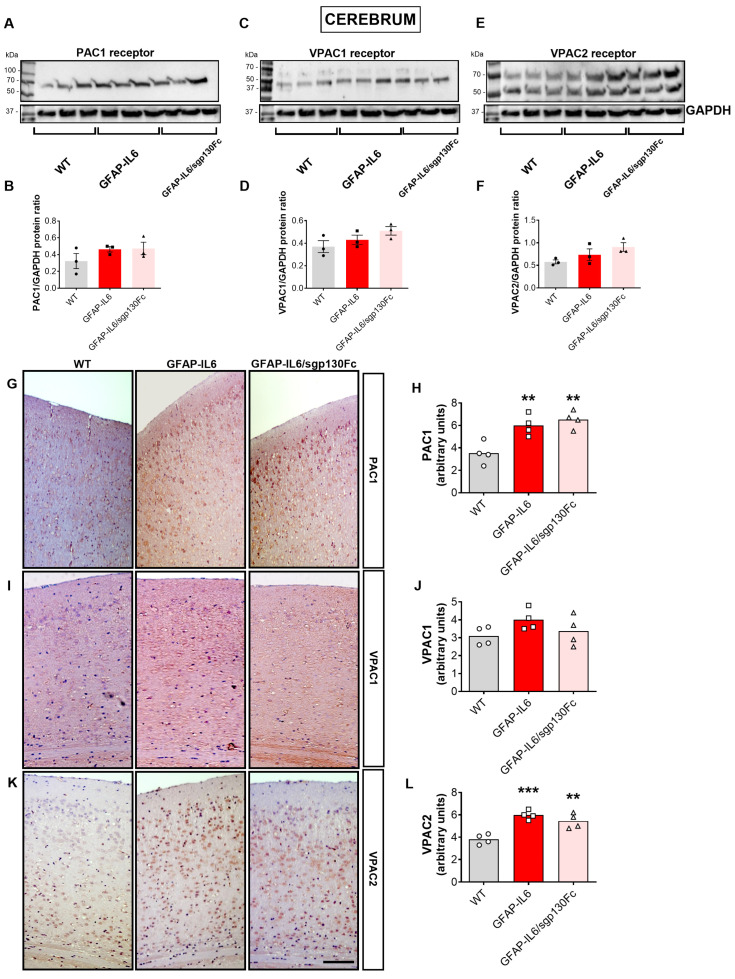
Expression of PAC1, VPAC1 and VPAC2 receptors in the cerebrum of GFAP-IL6 and GFAP-IL6/sgp130Fc mice. The presence of sgp130Fc does not prevent the increase in PACAP/VIP receptors in the cerebrum of GFAP-IL6 mice. (**A**,**C**,**E**) Western blots and (**B**,**D**,**F**) densitometry of bands obtained from lysates of cerebra from 6-month-old mice (n = 3 × genotype) that were separated by SDS-PAGE and quantified by NIH ImageJ software (version 1.52). GAPDH was used as the loading control. (**G**,**I**,**K**) PAC1, VPAC1 and VPAC2 immunohistochemistry was performed on paraformaldehyde fixed, paraffin-embedded sections (5 µm) of brains prepared from 6-month-old mice. Scale bar, 50 µm. (**H**,**J**,**L**) Semi-quantitative analyses of immunoreactivities were performed on at least three blinded sections per brain and on a minimum of four brains × genotype. Values represent the mean ± SEM with n = 4 brains per genotype. ** *p* < 0.01 or *** *p* < 0.001 vs. WT mice. One-Way ANOVA followed Tukey post-hoc test.

Based on our findings, demonstrating increased concentrations of PACAP and VIP peptides in the brain of GFAP-IL6 mice—only partly attenuated when trans-signalling was blocked—we sought to investigate the levels of PACAP/VIP receptors both in monogenic and bi-genic mutant mice. Immunoblots showed a slight but not significant increase in PAC1 in the cerebrum of GFAP-IL6 mice, which was not reduced in GFAP-IL6/sgp130Fc mice (Figure 4A,B, *F*_2,8_ = 1.452, not significant vs. WT). A similar pattern was also seen when interrogating protein levels of both the VPAC1 (Figure 4C,D) and VPAC2 receptors (Figure 4E,F).

Immunohistochemical analyses revealed more robust changes in PACAP/VIP receptors in the cerebral cortex of GFAP-IL6 and GFAP-IL6/sgp130Fc mice (Figure 4G–L). Specifically, PAC1 immunoreactivity (IR) was strongly increased both in GFAP-IL6 (Figure 4G,H, F_2,11_ = 12.50, ** *p* < 0.01 vs. WT) and GFAP-IL6/sgp130Fc mice (** *p* < 0.01 vs. WTs). In contrast, cortical VPAC1-IR did not show any statistically significant changes in either transgenic mouse (Figure 4I,J, *F_2,11_ = 1.901*, not significant vs. WT for both genotypes). VPAC2-IR in the cortex of GFAP-IL6 mice was remarkably increased (Figure 4K,L, *F*_2,11_ = 18.50, *** *p* < 0.001 vs. WT) and was only slightly diminished in double transgenic mice (** *p* < 0.01 vs. WT).

**Figure 5:**
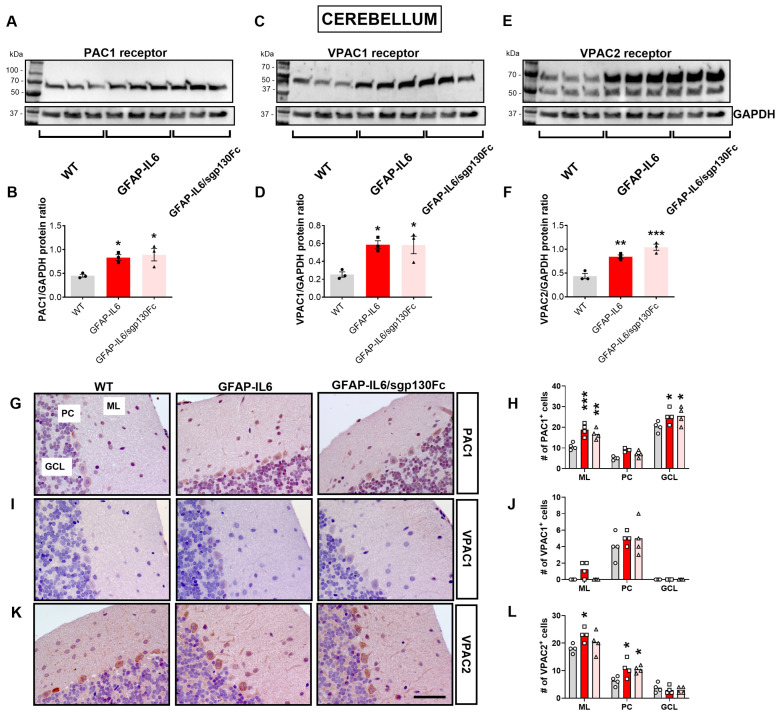
Expression of PAC1, VPAC1 and VPAC2 receptors in the cerebellum of GFAP-IL6 and GFAP-IL6/sgp130Fc mice. Inhibition of IL6 trans-signalling failed to reduce GFAP-IL6-driven induction of PACAP/VIP receptors in the mouse cerebellum. (**A**,**C**,**E**) Tissue lysates (15 µg protein per lane) from cerebellum of 6-month-old mice were subjected to SDS-PAGE followed by immunoblotting. (**B**,**D**,**F**) Quantification of band densities (n = 3 × genotype) by NIH ImageJ software. GAPDH was used as loading control. (**G**,**I**,**K**) PAC1, VPAC1 and VPAC2 immunohistochemistry was performed on paraformaldehyde fixed, paraffin-embedded sections (5 µm) of brains prepared from 6-month-old mice. Scale bar, 50 µm. (**H**,**J**,**L**) Stereological assessments of (**H**) PAC1^+^, (**J**) VPAC1^+^ and (**L**) VPAC2^+^ cells in each of the three cerebellar cortical layers was performed on at least three blinded sections per brain and using four brains × genotype. Values represent the mean ± SEM with n = 4 brains per genotype. * *p* < 0.05, ** *p* < 0.01 or *** *p* < 0.001 vs. WT mice. Western blots (**A**–**F**): One-Way ANOVA followed by Tukey post-hoc tests. Stereology (**G**–**L**): 2-Way ANOVA (factoring in both cell layers and genotypes) followed Tukey post-hoc tests. ML = Molecular layer; PC = Purkinje cells layer; GCL = Granular cell layer.

In the cerebellum, the effects of chronic IL-6 were more pronounced than in the cerebrum (Figure 5). Western blots of cerebellar protein lysates demonstrated a significant increase of PAC1 protein in GFAP-IL6 (Figure 5A,B, *F*_2,8_ = 7.696, * *p* < 0.05 vs. WT) and GFAP-IL6/sgp130Fc mice (* *p* < 0.05 vs. WT). Similarly, VPAC1 was increased in GFAP-IL6 mice cerebella (Figure 5C,D, *F*_2,8_ = 2.480, * *p* < 0.05 vs. WT) and was not affected by trans-signalling inhibition (* *p* < 0.05 vs. WT). VPAC2 protein showed a robust increase in both GL-IL6 (Figure 5E,F, *F*_2,8_ = 32.46, ** *p* < 0.01 vs. WT) and GFAP-IL6/sgp130Fc mice (*** *p* < 0.001 vs. WT).

To corroborate immunoblots findings, we conducted stereological analyses of PAC1^+^, VPAC1^+^ and VPAC2^+^ cells in each cerebellar layer of both monogenic and bi-genic mutant mice (Figure 5G–L). We report strong genotype-specific (*F*_2,27_ = 15.54, *p* < 0.0001) and cell layer-specific effects on the PAC1^+^ cell count (*F*_2,27_ = 121.8, *p* < 0.0001) but no genotype × cell layer interactions (*F*_4,27_ = 1.027, *p* = 0.4112). The number of PAC1^+^ cells was significantly increased in the molecular layer of both GFAP-IL6 (Figure 5G,H, *** *p* < 0.001 vs. WT) and GFAP-IL6/sgp130Fc mice (** *p* < 0.01 vs. WT) but not in the Purkinje cell layer (not significant vs. WT for both genotypes), although PAC1^+^ cells were marginally increased. In the granular layer, we also observed a significant increase of PAC1^+^ cells in both genotypes (Figure 5G,H, * *p* < 0.05 vs. WT for both genotypes).

For VPAC1, we did not detect any genotype-specific effects (*F*_2,27_ = 1.710, *p* = 0.1998) or genotype × cell layer interactions (*F*_4,27_ = 0.869, *p* = 0.8692), whereas cell layer-specific effects were significant (*F*_2,27_ = 80.78, *p* < 0.0001). VPAC1^+^ cells were scarce/absent in the molecular and granular layers of both mutant mice and WT littermates, whereas sparse VPAC1^+^ cells were found in the Purkinje cell layer, though no significant changes were seen when comparing the different genotypes (Figure 5I,J, not significant vs. WT).

Two-way ANOVA analyses of VPAC2^+^ cells demonstrated a significant genotype-specific effect (*F*_2,27_ = 5.012, *p* = 0.014) and a very strong cell layer-specific effect (*F*_2,27_ = 173.5, *p* < 0.0001), but no significant effects of genotype × cell layer interactions were found (*F*_4,27_ = 2.382, *p* = 0.076). The number of VPAC2^+^ cells was significantly increased in the molecular layer of GFAP-IL6 mice (Figure 5K,L, * *p* < 0.05 vs. WT) but not in GFAP-IL6/sgp130Fc mice (not significant vs. WT). Within the Purkinje cell layer, the VPAC2^+^ cell count was significantly increased both in monogenic and bi-genic mutant mice (* *p* < 0.05 vs. WT). Finally, only few cells were VPAC2^+^ in the granular layer, with no significant differences when comparing genotypes (not significant vs. WT).

## 3. Discussion

The data reported in this study is the first to demonstrate that CNS-restricted astrocyte-targeted IL-6 production enhanced the endogenous production of PACAP and VIP and increased the levels of the related receptors both in the cerebrum and, to a greater extent, in the cerebellum of GFAP-IL6 transgenic mice. In addition, by taking advantage of bi-genic mice forced to co-express both IL-6 and the sgp130Fc (GFAP-IL6/sgp130Fc) transgenes, we show that, despite trans-signalling playing a dominant role in modulating IL-6 driven activities of two main transcriptional members of the inflammasome, namely STAT3 and NFκB, its blockade fails to prevent most of the upregulation of PACAP and related receptors.

To date, previous studies on PACAP and VIP function in the CNS have largely contributed to establishing a protective role for these peptides [25,26]. PACAP/VIP beneficial activities in neurons can be either direct [27] or elicited indirectly through the ability of both peptides to stimulate glial cells to produce neurotrophic factors, such as activity-dependent neurotrophic factor (ADNF), activity-dependent neuroprotective protein (ADNP), neurotrophin-3, protease nexin-1 and RANTES [28,29,30,31]. Other indirect players mediating PACAP/VIP protection are inflammatory mediators, such as IL-1, IL-6 and macrophage inflammatory protein (MIP), which can be induced after CNS injury [28]. However, the ubiquitous distribution of PACAP and VIP and their receptors (PAC1, VPAC1-2) in glial [32,33], neuronal [34,35], immune [16,36] and endothelial cells [37] has led to the idea that these neuropeptides could also be implicated in controlling CNS homeostasis via neuro-immune modulatory activities. However, in vitro studies have identified divergent roles of the peptides on IL-6 release, with stimulatory activities in astrocytes [38] and inhibitory activities in microglia [21], making our understanding of peptides’ function in the immune system more complex.

Inspired by the idea of clarifying the forced production of IL-6 in astrocytes influenced the endogenous activity of the PACAP/VIP system and of establishing the consequences of trans-signalling inhibition on this neuroprotective system, we analysed the levels of both peptides and related receptors in the cerebrum and cerebellum of adult GFAP-IL6 mice and in animals co-expressing both IL-6 and the trans-signalling inactivator sgp130Fc (GFAP-IL6/sgp130Fc). Our findings showed that chronic IL-6 production in astrocytes was sufficient to increase PACAP/VIP levels, as well as receptors’ expression. Peptide concentration and the expression of PACAP/VIP receptors were higher in the cerebellum compared with the cerebrum of GFAP-IL6 mice, which is consistent with the higher levels of the transgene and the region-specific inflammatory milieu previously reported in these mice [5], which is likely due to the destruction of the cerebellar blood–brain barrier (BBB) and consequent macrophage infiltration. However, a blockade of trans-signalling largely failed to attenuate brain peptide concentrations, although VIP concentration was diminished both in the cerebrum and cerebellum. These results were paralleled by diminished activation of STAT3 and NFκB. Of note, the increase in Erk1/2^MAPK^ phosphorylation in GFAP-IL6 mice was not prevented by co-production of sgp130Fc, ruling out the potential involvement of this signalling cascade in IL-6 mediated inflammation. These outcomes, which are in apparent contradiction to the anti-inflammatory potential attributed to these two endogenous molecules, are in line with previous evidence indicating that local inflammation at various peripheral and CNS sites is associated with an upregulation of PACAP/VIP mRNAs [39,40,41]. In any case, assuming that inflammation may have been responsible for the induction of neuropeptides, a rationale to explain why VIP gene expression was induced in our model may be directly attributed to transgene expression [42]. Indeed, the IL-6 family of cytokines activate pathways that are known to regulate VIP gene transcription via STAT proteins and through gp130-responsive sites previously identified on the VIP gene [43]. Regarding PACAP, it is possible that gene expression is not directly regulated by resident glia but rather determined by the facilitated chemotactic recruitment of infiltrating lymphocytes at the site of inflammation, which can easily cross the disrupted BBB in these transgenic mice. This conclusion is based on earlier findings from a study in which SCID mice (which lack lymphocytes) underwent nerve axotomy, resulting in the suppression of PACAP gene induction. However, this suppression was reversed when the mice were pre-infused with splenocytes from WT animals. [44], providing evidence that infiltration of peripheral inflammatory cells may be required for the induction of PACAP expression.

The observed increases in PACAP and VIP levels, along with the upregulation of PAC1 and VPAC2 receptors in the cerebrum and cerebellum of GFAP-IL6 mice, suggest a complex regulatory mechanism at play, potentially reflecting the brain’s attempt to counteract chronic neuroinflammation. PACAP and VIP are neuropeptides known for their potent neuroprotective [29,45,46], anti-inflammatory [47,48,49] and immunomodulatory effects within the CNS [26,50]. Their upregulation in response to elevated IL-6 levels may represent an endogenous protective response aimed at mitigating neuroinflammatory damage. Specifically, PACAP’s ability to reduce cytokine production [51], inhibit apoptosis [52,53,54] and promote neuronal survival underscores its critical role in maintaining CNS homeostasis under stress conditions, such as those induced by chronic IL-6 expression. Similarly, VIP has been shown to modulate immune responses by inhibiting pro-inflammatory cytokine production and promoting the release of anti-inflammatory mediators [51,55,56]. The increased expression of PAC1 and VPAC2 receptors, which mediate the effects of PACAP and VIP respectively, suggests that the CNS is enhancing its sensitivity to these neuropeptides, likely in an effort to amplify their protective actions in the face of sustained inflammatory stimuli [26,57].

The physiological significance of these changes lies in their potential to influence a variety of CNS functions, including synaptic plasticity [58], neurogenesis [59] and the modulation of glial cell activity [60,61], all of which are crucial for maintaining neuronal integrity during inflammation [62]. However, the precise regulatory mechanisms driving these changes in PACAP, VIP and their receptors remain poorly understood, warranting further investigation. Such studies should focus on deciphering the signalling pathways involved and determining whether these changes represent a compensatory mechanism or a direct consequence of IL-6-driven pathology in neuroinflammatory conditions.

Overall, it is proposed that both PACAP and VIP are involved in the homeostatic control of the CNS during chronic inflammation. The marginal effect on the expression of neuropeptides (and receptors) in bi-genic GFAP-IL6/sgp130Fc mice does not contradict this hypothesis, as the blockade of trans-signalling does not completely abrogate IL-6-induced CNS inflammation and BBB leakage [5]. In addition, the same study reported that the expression of other IL-6 responsive genes, such as the suppressor of cytokine signalling 3 (*SOCS3*), was not reduced in mice that co-produced sgp130Fc. Therefore, we cannot exclude that the expression of PACAP and receptors may also be under the control of other pro-inflammatory mediators that persist when trans-signalling is inhibited or if induction occurs via the membrane-bound IL-6R (classic signalling).

While the study establishes a connection between IL-6 signalling and the expression of PACAP, VIP and their receptors, certain limitations warrant consideration. The investigation predominantly centres on the expression of PACAP/VIP and their receptors within the brain, yet it does not identify any specific glial cell populations implicated in these effects. Given that most PACAP/VIP receptor staining was localized to neurons, the role of various glial cells (e.g., astrocytes and microglia) in regulating the neuropeptide system under IL-6-induced inflammation remains unclear. Additionally, the study does not address the functional implications of the elevated PACAP/VIP levels and receptor expression with respect to neuroprotection or disease progression. Further research, possibly involving the exogenous administration of these neuropeptides in transgenic mice, may be necessary to elucidate their impact on neurodegenerative or neuroinflammatory outcomes, which is essential for understanding the physiological significance of the findings.

## 4. Materials and Methods

### 4.1. Animals

GFAP-IL6 and GFAP-IL6/sgp130Fc mice were generated and characterized as reported previously [5]. WT, GFAP-IL6 and GFAP-IL6/sgp130Fc bi-genic mice heterozygous for each transgene, were derived by interbreeding the GFAP-IL6 and GFAP-sgp130Fc parental lines. The genotype of all mice was verified by PCR analysis of tail DNA. All mice used in this study were fed ad libitum and were maintained under specific pathogen-free conditions. The rationale for our experimental design, including sample size and repetitions, was guided by previous findings in GFAP-IL6 mice, where a large effect size was observed [5]. Given the robust neuroinflammatory phenotype and significant changes in related targets like PACAP/VIP, we determined that small cohorts (n = 3–4 per group [WT, GFAP-IL6 and GFAP-IL6/sgp130Fc], respectively) would be sufficient to detect significant changes while adhering to the ethical principles of animal use. Approval for the use of mice in this study was obtained from the University of Sydney Animal Care and Ethics Committee (#758; approved on 02/2015), with Institutional Biosafety committee permits: 1. IBC ref #: 04N014 and IBC ref #: 07N011.

### 4.2. Antibodies

Immunoblotting was performed using antibodies raised in rabbits against STAT3 (1:1000), phospho-STAT3 (Tyr705, 1:2000), phospho-p44/42 MAPK (phospho-ERK 1/2) (Thr 202/Tyr 204, 1:3000), NFκB (p65, 1:3000) and phospho-NFκB (p65, Ser 536, 1:1000) that were from Cell Signaling (Danvers, MA, USA). Total ERK 1/2 antibody (1:10,000, host: rabbit) and GAPDH antibody (1:30,000, host: mouse) were from Sigma-Aldrich (Castle Hill, NSW, Australia). Horseradish peroxidase (HRP)-coupled-goat anti-rabbit IgG (SC2004, Santa Cruz Biotechnology Inc., Dallas, TX, USA) or goat anti-mouse IgG (Fc specific, A0168, Sigma-Aldrich) secondary antibodies were used for detection.

### 4.3. Enzyme-Linked Immunosorbent Assay (ELISA)

PACAP and VIP concentrations in the cerebrum and cerebellum of aged-matched six-month-old WT GFAP-IL6 and GFAP-IL6/sgp130Fc mice were determined using the commercially available PACAP and VIP mouse ELISA Kits (MyBioSource, San Diego, CA, USA, Cat no. MBS2503456 and MBS703048, respectively). Briefly, three mice from each group (n = 3) were euthanised by cervical dislocation, the brains were rapidly removed and the cerebra and cerebella were dissected. Tissue samples obtained were then immediately snap-frozen in liquid nitrogen and kept at −80 °C until needed. At the time of processing, wet tissues were weighed and diluted (1:10 wt/vol) in ice-cold phosphate buffered saline (PBS) containing 0.5% Tween-20 and a cocktail of freshly prepared protease and phosphatase inhibitors (1:100, Calbiochem, Sydney, Australia). Tissues were then homogenized on ice and cleared by centrifugation at 12,000× *g* rpm for 25′ at 4 °C. Finally, clarified lysates were assayed following the instructions given by the manufacturer. Optical density was measured at 450 nm using a microplate reader.

### 4.4. SDS–Polyacrylamide Gel Electrophoresis and Western Blotting

Tissues were homogenized in a RIPA lysis buffer (50 mM Tris-HCl (pH 7.5), 150 mM NaCl, 1 mM EDTA, 1% (wt/vol) deoxycholic acid, 1% (vol/vol) triton-X, 0.1% (wt/vol) sodium dodecyl sulfate (SDS), 2 mM PMSF, 50 mM NaF) containing freshly added protease and phosphatase inhibitor cocktails (1:100, Calbiochem, Sydney, Australia). Total protein levels were measured using the bicinchoninic acid (BCA) protein assay (Pierce, ThermoScientific, Ultimo, NSW, Australia). Lysates (15 μg) were fractionated by SDS-PAGE and analyzed by immunoblotting to determine the levels of STAT3, phospho-STAT3, ERK 1/2, phospho-ERK 1/2, NFκB, phospho-NFκB and GAPDH protein levels. Horseradish peroxidase (HRP)-coupled anti-rabbit or anti-mouse secondary antibodies and Immune star HRP detection kits (Bio-Rad, Gladesville, NSW, Australia) were used for detection. Of note, for the detection of PACAP/VIP receptors, gels were loaded with 30 μg of protein lysates. Detection was performed directly on membranes using the ChemiDoc Imaging system (Bio-Rad, Gladesville, NSW, Australia). Quantification of band intensities was performed using NIH Image-J, and the bands’ optical densities were normalized to that of the internal loading control glyceraldehyde 3-phosphate dehydrogenase (GAPDH).

### 4.5. Immunohistochemistry

Brains were fixed in 4% paraformaldehyde at 4 °C for 48 h before dehydration and embedding in paraffin. Then, 5 μm-thick coronal sections were cut with a microtome and mounted on glass slides (Sarstedt, Adelaide, SA, Australia). The sections were deparaffinised in xylene and rehydrated through decreasing concentrations of ethanol. Coronal sections included the cerebral cortex (cut at the level of the primary somatosensory area, ranging from −1.7 to −2.2 mm AP from bregma) or the cerebellum (−5.5 to −8.0 mm AP from bregma). Rehydrated sections were processed for immunolocalisation using the rabbit specific HRP/DAB (ABC) Detection IHC Kit (ab64261, Abcam, Melbourne, VIC, Australia), following the manufacturer’s protocol. Primary antibodies dilutions were as follows: PAC1 (1:500, GeneTex, Irvine, CA, USA; cat. No. GTX30026), VPAC1 (1:250, Sigma-Aldrich, SAB4503084) and VPAC2 (1:1000, Abcam, Melbourne, VIC, Australia, cat. No. ab2266). All primary antibodies were raised in rabbits. Hematoxylin was used as a counterstain to visualize the nuclei. After staining, sections were dehydrated in increasing concentrations of ethanol and xylene before being mounted. Images were taken on a ZEISS AxioScan.Z1 (Carl Zeiss, Australasia, NSW, Australia) at ×20 magnification.

In all cases, at least three tissue sections per animal were used. For staining quantification, several images (3–5) per section were taken, depending on the brain area. Since sections were counterstained with hematoxylin, the colour deconvolution plugin in ImageJ was used to separate DAB staining from hematoxylin counterstain. To perform stereological measurements in the cerebellum, PAC1^+^, VPAC1^+^ or VPAC2^+^ cells in each cerebellar layer (molecular, Purkinje and granule cell layers of each genotype) were counted blindly by at least two investigators using at least three regions of interest (200 × 200 μm = 40,000 μm^2^, each ROI area) per mouse and averaging cell counts.

### 4.6. Statistical Analyses

Statistical analyses were performed using GraphPad Prism version 8.00 for Windows, GraphPad Software, San Diego, CA, USA. For ELISA, RT-qPCRs and Western blots, comparisons among experimental groups were evaluated using a one-way analysis of variance (ANOVA) followed by a Tukey multiple comparison post hoc test. For the analyses of stereological data (cerebellum), two-way ANOVA (factoring in both cell layers and genotypes and their interactions) and Tukey post-hoc tests were used. Results are shown as mean ± standard error of the mean (SEM). *p* values of <0.05 were considered significant.

## 5. Conclusions

In conclusion, the current study identifies the PACAP/VIP neuropeptide system as an endogenous immune regulator, whose expression is increased in the CNS of mice with forced production of the pro-inflammatory cytokine IL-6 in astrocytes. A blockade of trans-signalling effectively reduced most of the pro-inflammatory pathways activated by the cytokine; however, it failed to reduce PACAP (but not VIP) production and prevented the upregulation of PACAP/VIP receptors. Considering these novel data, we suggest that IL-6-mediated induction of these protective neuropeptides (and receptors) may occur via the activation of classic IL-6 signalling and/or be secondary to the residual neuroinflammation in the brain of bi-genic mice. However, these specific aspects warrant further investigation. Moreover, given the association of elevated IL-6 levels with the pathogenesis of multiple sclerosis [63,64] and encephalomyelitis [65,66]—both characterized by demyelination and neurodegeneration—GFAP-IL6 mice may serve as a model to investigate certain neuroinflammatory aspects of these conditions. Chronic IL-6 elevation in astrocytes can lead to persistent neuroinflammation, gliosis and neuronal damage, which are key features of these diseases. Thus, understanding the dysregulation of the PACAP/VIP neuropeptide system in chronic neuroinflammation may pave the way for the development of novel therapeutic strategies for the treatment of MS and/or other relevant neurodegenerative diseases. In this context, future studies investigating which CNS cell subpopulations rely on trans-signalling vs. canonical IL-6 signalling are warranted.

## Data Availability

The raw data that support the findings of this study are available from the corresponding author upon reasonable request.
